# 

**Published:** 1968-10

**Authors:** 


### BRISTOL MEDICO-CHIRURGICAL JOURNAL

*(Incorporating the Medical Journal of the South-West)*

Established 1883

Vol. 83 (iii and iv) October, 1968 No. 308

### *Contents*

| | | Page |
|---|---|---|
| VOCATIONAL TRAINING FOR GENERAL PRACTICE .............. | *Michael Lennard* | 23 |
| A NURSE IN GENERAL PRACTICE .............. | *R. W. Morris* | 29 |
| FACTS AND FANCIES IN ORTHOPAEDICS .......................... | *Norman Capener* | 31 |
| A SHORT HISTORY OF ISOLATION ACCOMMODATION IN BRISTOL ........... | *D. W. Wright* | 35 |
| NEWLY APPOINTED CONSULTANTS ............................ | | 38 |
| NOTES & NEWS ............................................................ | | 39 |

*PUBLISHED QUARTERLY*

*ANNUAL SUBSCRIPTION* **16/-** *POST FREE*

### BRISTOL MEDICO-CHIRURGICAL SOCIETY

President 1967-1968 : Prof. T. F. Hewer.

President-elect : Mr. A. L. Eyre-Brook.

Honorary Secretary : Dr. A. T. M. Roberts, 8 Harley Place, Bristol 8.

Honorary Treasurer : Dr. J. Macrae, Ham Green Hospital, Pill.

The Society was founded in 1874. In 1883 publication of this Journal began, and has continued quarterly since then. During the years 1949 to 1962 it appeared under the title of "The Medical Journal of the South West".

The Society founded a medical library in 1890, which in 1892 was combined with that of the Bristol Medical School. This became the University of Bristol Medical Library in 1925, and an agreement in that year confirmed the privilege of Members to use the University Medical Library.

### THE BRISTOL MEDICO-CHIRURGICAL JOURNAL

*Editorial Committee*

Dr. N. J. Brown, Southmead Hospital, Bristol. *Joint Editor.*

Dr. A. B. Raper, Department of Pathology, Bristol Royal Infirmary. *Joint Editor.*

Dr. J. Macrae, Ham Green Hospital, Pill, Bristol. *Honorary Treasurer.*

Dr. R. C. Wofinden, Central Clinic, Bristol.

Mr. A. E. S. Roberts, Medical School Library, University Walk, Bristol 8. *Secretary.*

### NOTICE TO CONTRIBUTORS

The Journal is especially concerned to provide a place for the recording of medical thought, interests, and practice in and around Bristol. It therefore welcomes articles that, as well as being of immediate interest to members, will document the local and contemporary medical scene for our successors.

Original articles are invited provided that they have not been published elsewhere. References in the text should be by author's name, with year of publication in parenthesis; they should be listed at the end in alphabetical order. Illustrations should be supplied ready for reproduction. Line drawings should be in indian ink on firm white paper or board. The author will be informed if it is found necessary to ask him to pay for the making of blocks. The Editoral Committee reserves the right to make literary corrections.

The following contributions will be especially welcome: notes of forthcoming events, meetings held, degrees conferred, staff appointments, honours and public appointments and other local news.

### NOTES AND NEWS

Professor C. Bruce Perry has been appointed Lumleian Lecturer for 1969 at the Royal College of Physicians of London.

The Jacksonian Prize of the Royal College of Surgeons of England has been awarded to Mr. J. H. Peacock.

Mr. H. J. Espiner has been appointed consultant general surgeon, United Bristol Hospitals and South Western Regional Hospital Board.

University of Bristol Examination results :—

| | |
|---|---|
| M.D. | J. R. Clamp, B. P. Harrold, C. E. Polkey, M. A. Voyce, C. B. Williams. |
| Ch.M. | A. H. C. Ratliff. |
| Ph.D. | P. G. G. Darke, S. I. Egglestone, A. Livingston, M. S. A. Talukder, Marjorie M. Wedgewood Bunyard. |
| D.P.H. | June M. Brown, S. I. Cobein, Caryl R. Gibbs, Lillian S. N. A. Khan, H. K. Menokpor, C. J. Padfield, Lilian J. Powell, Dorothy M. H. Roberts, Pauline M. Seymour-Cole, E. E. Warr. |
| M.B., Ch.B. | Margaret A. S. Morton (with honours), R. S. E. Wilson (with honours), E. K. O. Ajai-Ajagbe, B. Attock, B. G. Bowden, A. V. J. Butler, Jill Carah, D. A. Coates, R. A. Cowper, Gillian D'Aeth, R. H. Diprose, W. M. Donovan, Sandra M. Egan, S. A. Fairham, M. G. W. Fisher, G. C. H. Freakley, Marilyn D. Gibbs, Irene M. G. Gregory-Smith, R. A. Haward, Judith M. Hayes, M. R. Hetley, I. R. Lanman, D. T. McCarthy, D. J. Morris, R. A. Mountford, Ruth G. E. Philpott, Rachel G. Pinniger, A. K. Pridie, Angela M. Shepherd, Rosemary D. Sheppard, Hazel J. Skinner, G. R. Swingler, Enid J. Taylor, D. J. Thornton, C. A. Westwood, B. F. Whyte, B. S. Yap. |

